# A rare genetic variant of *BPIFB4* predisposes to high blood pressure via impairment of nitric oxide signaling

**DOI:** 10.1038/s41598-017-10341-x

**Published:** 2017-08-29

**Authors:** Carmine Vecchione, Francesco Villa, Albino Carrizzo, Chiara Carmela Spinelli, Antonio Damato, Mariateresa Ambrosio, Anna Ferrario, Michele Madonna, Annachiara Uccellatore, Silvia Lupini, Anna Maciag, Larisa Ryskalin, Luciano Milanesi, Giacomo Frati, Sebastiano Sciarretta, Riccardo Bellazzi, Stefano Genovese, Antonio Ceriello, Alberto Auricchio, Alberto Malovini, Annibale Alessandro Puca

**Affiliations:** 1 IRCCS Neuromed, 86077 Pozzilli (IS), Italy; 2 Department of Medicine and Surgery, University of Salerno, Fisciano, 84084 (SA) Italy; 3 Cardiovascular Research Unit, IRCCS MultiMedica, 20099 Sesto San Giovanni (MI), Italy; 4 Institute of Biomedical Technologies, National Research Council, 20090 Segrate (MI), Italy; 5 University of Milan, Via Festa del Perdono, 20122 Milan, Italy; 6 Department of Translational Research and New Technologies in Medicine and Surgery, University of Pisa, Pisa, 56126 Italy; 7 Department of Medico-Surgical Sciences and Biotechnologies, Sapienza University of Rome, 04100 Latina, Italy; 8 Laboratory of Informatics and Systems Engineering for Clinical Research, Istituti Clinici Scientifici Maugeri, 27100 Pavia, Italy; 9 Department of Electrical, Computer and Biomedical Engineering, University of Pavia, Pavia, Italy; 10 Diabetes Endocrine and Metabolic Diseases Unit, IRCCS MultiMedica, 20099 Sesto San, Giovanni (MI) Italy; 11 Institut d’Investigacions Biomèdiques August Pi i Sunyer (IDIBAPS) and Centro de Investigación Biomedica en Red de Diabetes y Enfermedades Metabólicas Asociadas (CIBERDEM), Barcelona, Spain; 12 Department of Cardiovascular and Metabolic Diseases, IRCCS MultiMedica, 20099 Sesto San, Giovanni (MI) Italy; 13 TIGEM (Telethon Institute of Genetics and Medicine), 80078 Pozzuoli, Italy; 14 Department of Translational Medicine, “Federico II” University, Napoli, Italy

**Keywords:** Genetic association study, Molecular medicine

## Abstract

*BPIFB4* is associated with exceptional longevity: four single-nucleotide polymorphisms distinguish the wild-type form from a longevity-associated variant conferring positive effects on blood pressure. The effect of a rare variant (RV; allele frequency, 4%) on blood pressure is unknown. Here, we show that overexpression of RV-BPIFB4 in *ex-vivo* mouse vessels impairs phosphorylation of endothelial nitric oxide synthase (eNOS), blunting acetylcholine-evoked vasorelaxation; *in vivo*, virally mediated overexpression of RV-BPIFB4 increases blood pressure, an action absent in eNOS-deficient mice. In humans, we found RV carriers to have increased diastolic blood pressure, a finding that was more marked in subjects on anti-hypertensive medication; moreover, recombinant RV-BPIFB4 protein impaired eNOS function in *ex-vivo* human vessels. Thus, RV-BPIFB4 acts directly on blood pressure homeostasis and may represent a novel biomarker of vascular dysfunction and hypertension.

## Introduction

There is tight correlation between exceptional longevity and integrity of the cardiovascular system. An example is the fine-tuning of blood pressure: this is lost during aging, becoming a strong risk factor for disability and mortality. Indeed, reduced activity during aging of endothelial nitric oxide synthase (eNOS) – a crucial enzyme for endothelial integrity and function – leads to increased susceptibility to cardio- and cerebro-vascular diseases^1–4^. The importance of eNOS is underscored by the fact that caloric restriction – which protects the cardiovascular system and extends life-span – is inefficient in a context of *eNOS* gene knock-down^5^.

Long-living individuals (LLIs) carry a favorable genetic profile characterized by an enrichment of alleles that protect from aging-related and cardiovascular diseases^6, 7^. One of these alleles is the minor allele *rs2070325* (I229V) of bactericidal/permeability-increasing fold-containing family B member 4 (*BPIFB4*), which we found enriched as a homozygous trait in LLIs^8^. *rs2070325* is part of a four-SNP (single-nucleotide polymorphism) haplotype that distinguishes the wild-type (WT) Ile229/Asn281/Leu488/Ile494-BPIFB4 isoform (allele frequency, 66%) from the longevity-associated variant (LAV) Val229/Thr281/Phe488/Thr494-BPIFB4 (allele frequency, 29.5%); a rare variant (RV) Ile229/Asn281/Phe488/Thr494-BPIFB4 – which retains only the third and fourth amino acid substitutions of the LAV– is also present (allele frequency, 4%).

Homozygous (a/a) *rs2070325* individuals have significantly more eNOS activated in their mononuclear cells than do heterozygous (a/A) or WT (A/A) carriers^8^, and healthily aged LLIs have significantly higher serum BPIFB4 than do frail individuals^9^. In a murine model of mesenteric artery function – involving adeno-associated virus 9-mediated overexpression of a BPIFB4 isoform *in vivo* and subsequent assessment of vascular functionality *ex vivo* – the LAV potentiated eNOS activity and endothelial function, reducing blood pressure levels; no effect was observed with WT-BPIFB4. Furthermore, overexpression of LAV-BPIFB4 improved stem-cell homing and revascularization in a model of limb ischemia. Compared to WT-BPIFB4, the LAV was more cytoplasmic, more phosphorylated at serine 75 by the protein kinase R–like endoplasmic reticulum kinase (PERK), and was more efficiently bound to protein 14-3-3, forming a complex needed for the recruitment of heat shock protein (HSP)90 and eNOS activation^8^. In that study, the effect of the RV was not determined.

Here, we show that overexpression of the RV in WT mice impairs eNOS signaling and endothelial function, evoking an increase in blood pressure. Coherently, no effects were observed in eNOS-deficient mice. Of note, we found in humans that RV-*BPIFB4* carriers had significantly increased blood pressure. Moreover, exposure of *ex-vivo* human vessels to a recombinant RV-BPIFB4 protein negatively modulated endothelial function and eNOS activation, corroborating the findings in mice.

## Results

### Overexpression of RV-BPIFB4 in *ex-vivo* mouse vessels impairs endothelial function and eNOS activation

**Figure 1:**
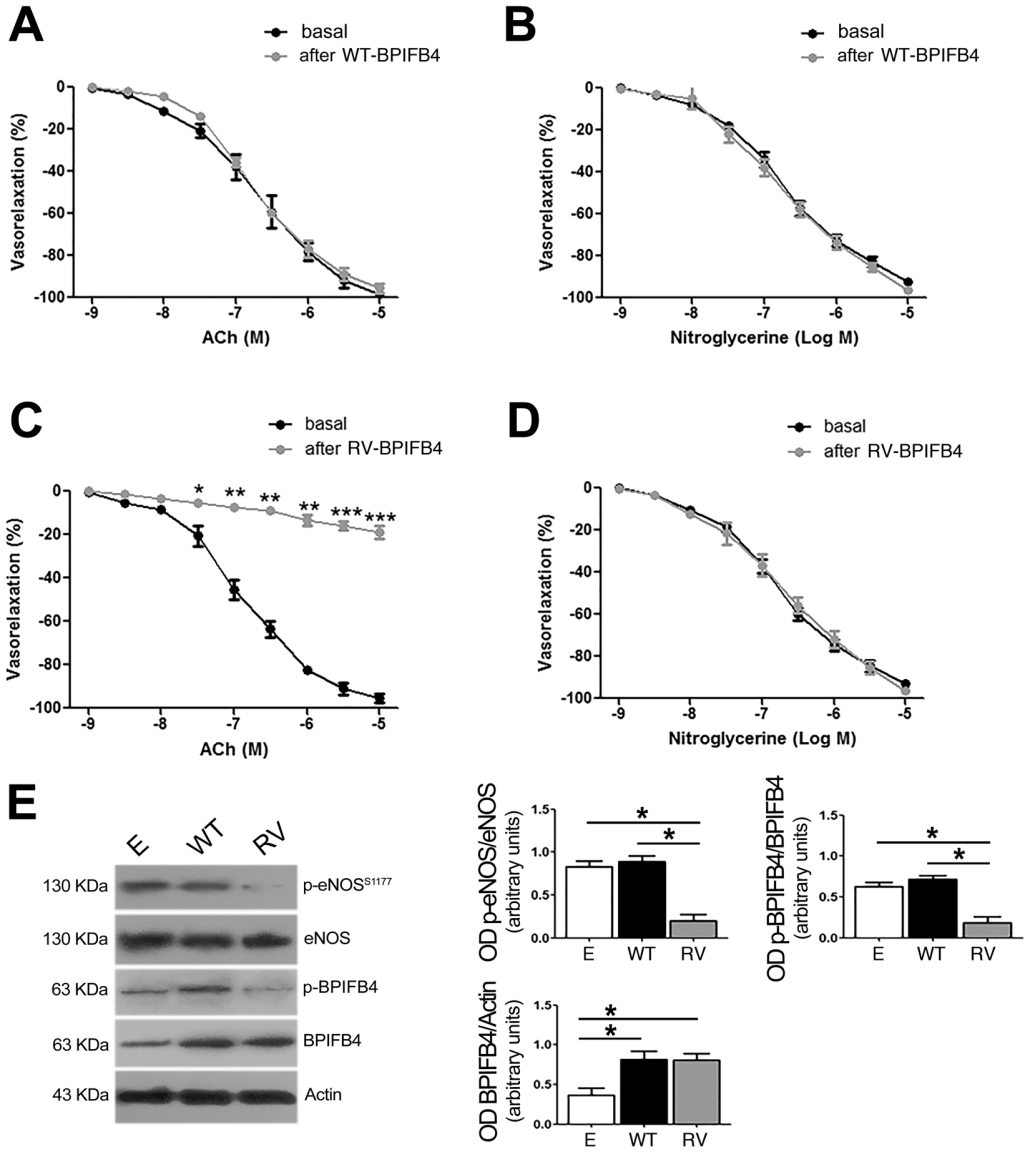
Response of *ex vivo* mouse mesenteric arteries to transfection with RV-*BPIFB4*. (**A**,**B**) Graphs of vascular response to increasing doses of acetylcholine (ACh) or nitroglycerine in mouse mesenteric arteries before and after transfection with plasmids encoding WT-*BPIFB4*. (**C**,**D**) Graphs of vascular response to increasing doses of acetylcholine (ACh) or nitroglycerine in mouse mesenteric arteries before and after transfection with plasmids encoding RV-*BPIFB4* (n = 7 for each experiment). *p < 0.05; **p < 0.01;***p < 0.001 (two-way ANOVA followed by Bonferroni post hoc test). (**E**) *left*, Representative immunoblot of mesenteric artery lysate collected after vascular reactivity studies; *right*, Optical density measurements of immunoblottings. Columns are the mean ± SEM of 3 independent experiments. *p < 0.05 (one-way ANOVA analysis followed by Bonferroni post hoc test). Images were cropped using Adobe Photoshop, full-length blots are presented in Supplementary Fig. 2.

We first explored whether the RV modulated vascular tone in an experimental model. To this end, we transfected *ex-vivo* mouse mesenteric arteries with plasmids encoding either RV-*BPIFB4* or the WT isoform. Overexpression of RV-BPIFB4 impaired acetylcholine-evoked vasorelaxation (Fig. 1A–D) and eNOS phosphorylation at serine 1177 (Fig. 1E), the main activation site of the enzyme. Moreover, phosphorylation of BPIFB4 at serine 75 – which is needed to recruit 14-3-3 and activate eNOS-dependent production of NO – was significantly reduced by overexpression of RV-BPIFB4 (Fig. 1E).

### Administration of AAV-RV-*BPIFB4* increases blood pressure in mice

**Figure 2:**
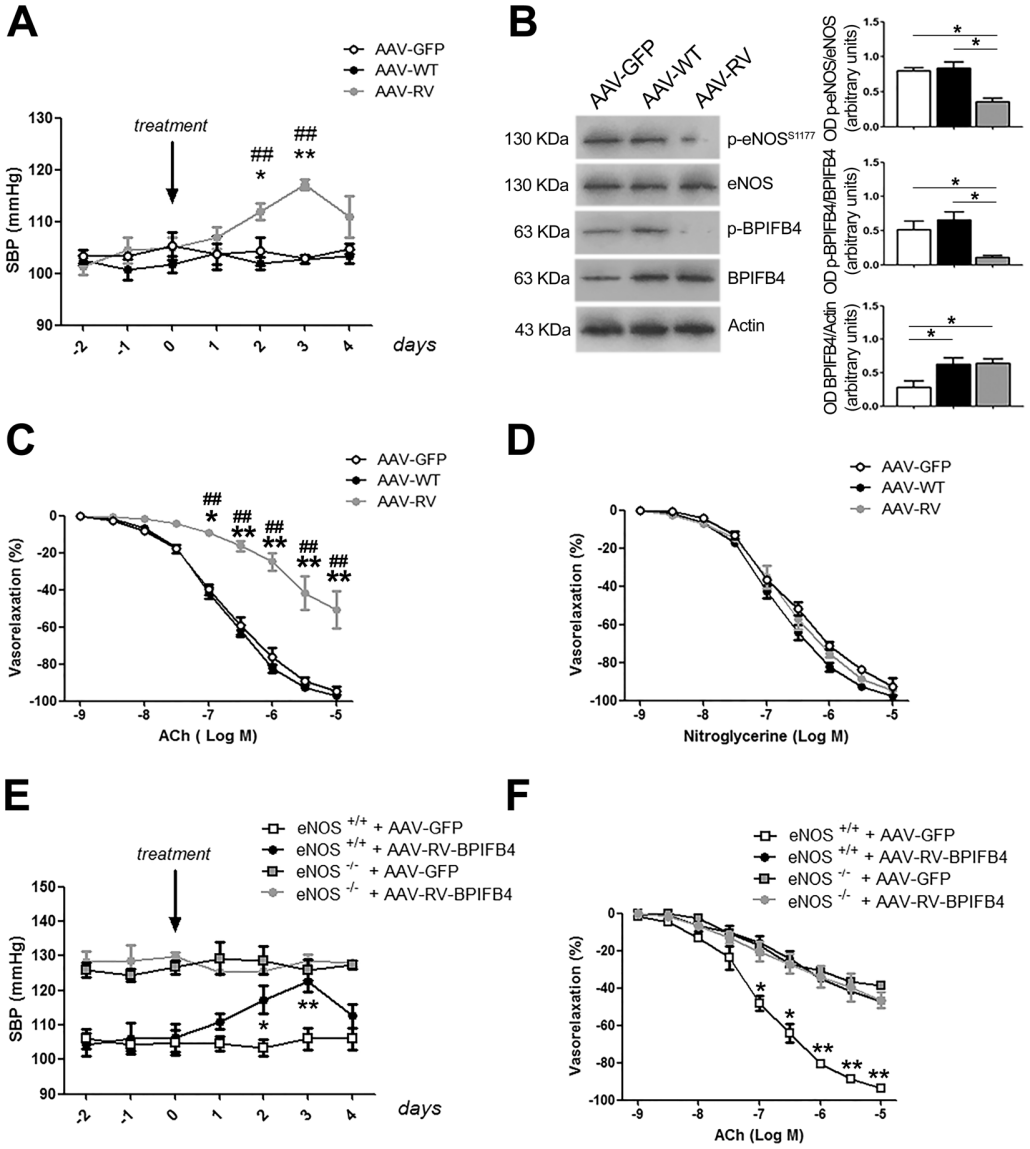
*In vivo* infection of mice with RV-BPIFB4. (**A**) Graph of systolic blood pressure (SBP) in C57BL/6 mice infected with AAV vectors encoding WT-*BPIFB4*, RV-*BPIFB4*, or green fluorescent protein (*GFP*). The arrow indicates injection of AAV in the femoral artery (day 0). Data are mean ± SEM (n = 6/group). *p < 0.05; **p < 0.01 vs. AVV-WT-BPIFB4; ^##^p < 0.01 vs. AVV-GFP (two-way ANOVA followed by Bonferroni post hoc test). (**B**) representative immunoblottings (*left*) and optical density measurements (*right*) of mesenteric arteries harvested from infected mice. Columns are the mean ± SD of 3 independent experiments.*p < 0.05 (one-way ANOVA analysis followed by Bonferroni post hoc test). Images were cropped using Adobe Photoshop, full-length blots are presented in Supplementary Fig. 3. (**C**,**D**) *ex-vivo* response of mesenteric arteries removed from mice after infection with AAV-WT-*BPIFB4*, AAV-RV-*BPIFB4*, or AAV-*GFP* to increasing doses of acetylcholine (ACh) or nitroglycerine. Two-way ANOVA, in combination with Bonferroni post-tests. (**E**) Graph of systolic blood pressure (SBP) in eNOS knock-out mice (*eNOS*^−/−^) and wild-type mice (*eNOS*^+/+^) infected with AAV vectors encoding RV-*BPIFB4* or green fluorescent protein (*GFP*). The arrow indicates injection of AAV in the femoral artery (day 0). Data are mean ± SEM (n = 5/group). *p < 0.05; **p < 0.01 vs. AVV-*GFP* + *eNOS*^+/+^ (two-way ANOVA followed by Bonferroni post hoc test). (**F**) Graph of ex-vivo vascular response in mouse mesenteric arteries removed from wild-type C57BL/6 (*eNOS*^+/+^) and eNOS knock-out (*eNOS*^−/−^) mice infected with AAV-RV-*BPIFB4* or AAV-*GFP*, to increasing doses of acetylcholine (ACh). Data are presented as mean ± SEM. *p < 0.05; **p < 0.01 vs. all (two-way ANOVA followed by Bonferroni post hoc test).

To evaluate the *in-vivo* relevance of the findings obtained through plasmid transfection, we generated RV- and WT-*BPIFB4*-encoding adeno-associated viral vectors (AAV; serotype 9 with a TBG promoter), and administered them to normotensive mice through the femoral artery. Compared to control, AAV-RV-*BPIFB4* significantly increased systolic blood pressure (SBP) two days after infection; SBP reached a maximum at day 3 and returned to a level that was not significantly different from baseline at day 4 (Fig. 2A). AAV-WT-*BPIFB4* did not elicit any effect on blood pressure, as reported previously by us^8^.

Analyses of the mesenteric arteries harvested after blood pressure measurements showed that phosphorylation on eNOS and BPIFB4 was impaired in vessels of mice administered AAV-RV-*BPIFB4* (Fig. 2B); coherently with what we observed with ex-vitro plasmid transfections, acetylcholine-elicited endothelial vasorelaxation was blunted in these vessels, whereas that elicited by nitroglycerine was not (Fig. 2C,D).

To definitively define the role of NO in the negative effect of RV-BPIFB4 on vascular homeostasis, we performed experiments on eNOS-deficient mice, which present with higher blood pressure levels when compared to WT counterparts. In this experimental setting, transduction with AAV-RV-*BPIFB4* failed to influence SBP **(**Fig. 2E**)**. Of note, the SBP of WT mice transduced with AAV-RV-*BPIFB4* rose to a level that was not significantly different to the baseline in eNOS-deficient mice. At the functional level, ex-vivo vascular reactivity studies showed that infection with AAV-RV-*BPIFB4* impaired acetylcholine-evoked vasorelaxation in WT vessels to a similar extent to that inherent in eNOS-deficient ones **(**Fig. 2F**)**. These data clearly demonstrate that impairment of NO is responsible for the negative effect of RV-BPIFB4 on vascular function and blood pressure.

### RV-BPIFB4 is associated *with* high blood pressure in humans

In the Pilot 1 data set of the 1000 Genomes Project–Coriell Institute for Medical Research (http://www.1000genomes.org/category/phase-1/), the *rs2070325* (Ile229Val) variation of BPIFB4 (identifier: P59827.2) is strongly correlated with *rs2889732* (Asn281Thr) (r^2^ = 0.93; D′ = 1); both variations show a limited amount of recombination events with *rs11699009* (Leu488Phe) and *rs11696307* (Ile494Thr) (r^2^ > 0.85; D′ > 0.92). Therefore, the three main alternative haplotypes of *BPIFB4* are the WT (Ile229/Asn281/Leu488/Ile494-BPIFB4), LAV (Val229/Thr281/Phe488/Thr494-BPIFB4), and RV (Ile229/Asn281/Phe488/Thr494-BPIFB4) isoforms, which carry the major alleles *rs2070325* (A) and *rs2889732* (A) and the minor alleles *rs11699009* (T) and *rs11696307* (C). Thus, we genotyped for *rs2070325* and *rs11699009* to determine the haplotype of 461 consecutive individuals that had been enrolled for an epidemiology study on diabetes. The clinical characteristics of the cohort are reported in Table 1. Supplementary Table 1 gives the association of the considered clinical variables with diastolic and systolic blood pressures.

**Table 1:** Clinical Characteristics of Enrolled Patients.

Characteristic	Value (n = 461)
Sex	
Male	194 (42.08%)
Female	267 (57.92%)
Age (years)	62 (55–69); 61.18 ± 8.78
Diabetic status	
Non-diabetic	239 (51.96%)
Pre-diabetic	164 (35.65%)
Diabetic	57 (12.39%)
Diastolic blood pressure	80 (70–85); 76.63 ± 11.93
Systolic blood pressure	130 (120–140); 128.69 ± 15.33
Glucose tolerance test (FPG)	89 (81–96); 86.69 ± 12.19
Glucose tolerance test (2HPG)	112.5 (91–138); 120.11 ± 42.45
Body mass index	26.40 (23.85–29.30); 27.01 ± 4.56
Triglyceridemia	99 (76–138); 115.85 ± 64.71
Total cholesterolemia	205.5 (180–229); 206.13 ± 35.68
High density lipoprotein cholesterolemia	56 (46–65); 56.84 ± 15.01
Anti-hypertensive treatment	
Yes	182 (60.52%)
No	279 (39.48%)

Notes: FPG, fasting plasma glycemia; 2HPG, 2-hour plasma glycemia (after ingestion of 75 g glucose). Distributions are described by median (interquartile range); mean ± SD or frequency (%).

The two tested SNPs (*rs2070325* and *rs11699009*) were characterized by intermediate to strong evidence of linkage disequilibrium (r^2^ = 0.80; D′ = 0.96); neither deviated significantly from the Hardy–Weinberg equilibrium (HWE, p > 0.05), with missing data fractions corresponding to 4% and 6%, respectively. The allele frequencies of the analyzed variants and deriving haplotype alleles are reported in Table 2. A total number of 418 subjects (91%) had no missing values for the two SNPs and for SBP and DBP measurements.

**Table 2:** SNPs and Haplotype Allele Frequencies^1^.

Variant	Allele	Frequency
*rs2070325*	A, G	G (0.348), A (0.652)
*rs11699009*	C, T	T (0.381), C (0.619)
Haplotype		
LAV	*rs2070325* = G; *rs11699009* = T	0.337
WT	*rs2070325* = A; *rs11699009* = C	0.613
RV	*rs2070325* = A; *rs11699009* = T	0.042
Other	*rs2070325* = G; *rs11699009* = C	0.008

^1^Complete data on both SNPs, SBP, and DBP was obtained in 418/461 subjects (91%).

The association of both SNPs with DBP and SBP traits was tested assuming a recessive model (*rs2070325*: GG vs. GA/AA; and *rs11699009*: TT vs. TC/CC). Since the LAV is represented by the minor allele of the two SNPs in haplotypic phase, a recessive model was tested for this allele too (i.e., LAV homozygotes – defined as *rs2070325* = G and *rs11699009* = T on both chromosomes – vs. LAV heterozygotes – defined as *rs2070325* = G and *rs11699009* = T on one chromosome – plus remaining haplotype carriers pooled). For the remaining two haplotypes with frequency >1% (i.e., RV and WT), dominant and recessive genetic models were assumed (i.e., for RV, RV haplotype carriers – defined as *rs2070325* = A and *rs11699009* = T on at least one chromosome – vs. non-carriers; and for WT, WT homozygotes – defined as *rs2070325* = A and *rs11699009* = C on both chromosomes – vs. WT heterozygotes – defined as *rs2070325* = A and *rs11699009* = C on one chromosome – plus remaining haplotype alleles pooled, respectively). Of note, less than 1% of patients were heterozygous carriers for *rs2070325* and WT at *rs11699009*, so we can assume that all individuals heterozygous for both SNPs are *bona fide* heterozygous carriers of the LAV because the chance of finding a compound heterozygous for *rs2070325* and *rs1699009* is <1/1,000.

Of the two tested variants, individuals homozygote for the minor allele *rs2070325* shared a statistically significant reduction in terms of DBP when compared to major allele carriers (Table 3). No statistically significant impact of the SNP was observed on SBP (Table 4). Similarly, individuals homozygote for the LAV haplotype had a statistically significant reduction in terms of DBP when compared to the rest of the cohort. No association between the LAV and SBP was observed.

**Table 3:** Impact of SNPs and haplotypes on diastolic blood pressure.

	Genotype or haplotype allele		Set	Count		Median (IQR); Mean ± SD - SBP (mm Hg)		P
	Ref.	Baseline		Ref.	Baseline	Ref.	Baseline	
SNP								
*rs2070325*	GG	GA/AA	All	46	394	70 (60–80); 73.59 ± 14.78	80 (70–85); 76.99 ± 11.54	0.0259*
*rs11699009*	TT	TC/CC	All	54	379	70 (60–90); 75.46 ± 14.99	80 (70–85); 76.79 ± 11.53	0.2772
haplotype								
LAV	LAV homo	LAV carriers/other	All	41	377	70 (60–80); 73.54 ± 14.76	80 (70–85); 76.9 ± 11.67	0.0386*
WT	WT homo	WT carriers/other	All	149	269	80 (70–80); 76.17 ± 11.77	80 (70–85); 76.8 ± 12.19	0.5652
RV	RV carriers	other	All	34	384	70 (76–90); 81.47 ± 12.22	80 (70–80); 76.14 ± 11.93	0.0132*
			On therapy	12	154	90 (80–100); 89.17 ± 9.25	80 (70–80); 76.71 ± 11.27	0.0007*
			No therapy	22	230	80 (70–88.75); 77.27 ± 11.72	80 (70–80); 75.76 ± 12.37	0.5102

SNP/Haplotype = SNP or haplotype allele; Ref. = reference, effect genotype or haplotype allele; Baseline = baseline genotype or haplotype; set = subset of the cohort analyzed; Count = genotypes or alleles count; Median (IQR); Mean ± SD = median (25^th^, 75^th^ percentiles) and mean ± standard deviation diastolic blood pressure by genotype or allele; p = p-value from the Wilcoxon rank sum test. *p-value < 0.05.

**Table 4:** Impact of SNPs and haplotypes on systolic blood pressure.

	Genotype or haplotype allele		Set	Count		Median (IQR); Mean ± SD - SBP (mm Hg)		P
	Ref.	Baseline		Ref.	Baseline	Ref.	Baseline	
SNP								
*rs2070325*	GG	GA/AA	All	46	394	120 (110–140); 124.57 ± 15.73	130 (120–140); 128.9 ± 15.20	0.0993
*rs11699009*	TT	TC/CC	All	54	379	130 (110–140); 126.76 ± 17.70	130 (120–140); 128.94 ± 15.12	0.5825
haplotype								
LAV	LAV homo	LAV carriers/other	All	41	377	130 (110–140); 124.88 ± 15.83	130 (120–140); 128.79 ± 15.30	0.1906
WT	WT homo	WT carriers/other	All	149	269	130 (120–140); 127.89 ± 14.98	130 (120–140); 128.69 ± 15.61	0.4990
RV	RV carriers	other	All	34	384	130 (120–150); 133.38 ± 18.82	130 (120–140); 127.96 ± 14.98	0.0670
			On therapy	12	154	150 (137–150); 146.25 ± 15.54	130 (120–140); 130.68 ± 15.67	0.0015*
			No therapy	22	230	125 (120–138); 126.36 ± 16.85	130 (116–140); 126.14 ± 14.25	0.9182

SNP/Allele = SNP or haplotype allele; Ref. = reference, effect genotype or haplotype allele; Baseline = baseline genotype or haplotype; set = subset of the cohort analyzed; Count = genotypes or alleles count; Median (IQR); Mean ± SD = median (25^th^ and 75^th^ percentiles) and mean ± standard deviation systolic blood pressure by genotype or allele; p = p-value from the Wilcoxon rank sum test. *p-value < 0.05.

RV carriers were characterized by significantly increased DBP and by a borderline increase in SBP (Tables 3 and 4). Stepwise linear regression, with a combination of forward search and backward elimination, starting from the full set of informative demographical and clinical covariates (see Methods section), confirmed that the RV modulated DBP even when accounting for potential confounders (Beta, 4.98 mm Hg; 95% CI, 1.15–8.81; p = 0.011) (Supplementary Table 2). Stepwise linear regression including the RV x anti-hypertensive treatment interaction term revealed a specific, increasing impact of the haplotype on DBP (interaction, p = 0.006) and on SBP (interaction, p = 0.007) in individuals under treatment, accounting for informative demographical and clinical covariates. Further analysis of the subgroup of patients taking medication for hypertension (n = 166) also showed that the presence of the rare haplotype variant had an increased effect in this subset (Tables 3 and 4). Multivariate stepwise linear regression including informative covariates confirmed the impact of the RV on DBP and SBP in this subgroup of individuals (DBP: Beta, 11.54 mm Hg; 95% CI, 52–17.56; p = 0.0002. SBP: Beta, 13.19 mm Hg; 95% CI, 4.71–21.66; p = 0.003). No statistically significant differences in DBP or SBP were observed between RV carriers and non-carriers in the subgroup of 252 individuals not on anti-hypertensive medication. Of note, anti-hypertensive treatment did not differ in RV carriers vs. non-carriers, and did not significantly influence the association results (Supplementary Table 3).

We then compared the distribution of SBP and DBP in LAV homozygotes, RV carriers, and WT homozygotes within the whole cohort and within subgroups stratified on the basis of treatment (Supplementary Fig. 1). Mean DBP was significantly higher in the RV carrier group vs. the LAV homozygote and WT homozygote groups (p = 0.009 and p = 0.02, respectively), with more marked differences between the subgroups of patients taking anti-hypertensive drugs (p = 0.004 and p = 0.003, respectively). The RV carrier group was also characterized by a significantly increased mean SBP vs. the LAV homozygote group (p = 0.044), but not vs. the WT homozygote group (p = 0.074); again the differences were greater between subgroups on anti-hypertensive drugs (RV vs. LAV homozygotes, p = 0.002; and RV vs. WT homozygotes, p = 0.01).

### Exposure to recombinant RV-BPIFB4 modulates the reactivity of human vessels

To assess whether the results obtained in the experimental models were relevant to the human setting, we performed ex-vivo vascular reactivity studies on superior thyroid arteries removed from patients undergoing carotid revascularization. We chose vessels with the best endothelial vasodilation, evaluating the effects of RV- and WT-BPIFB4 recombinant proteins.

**Figure 3:**
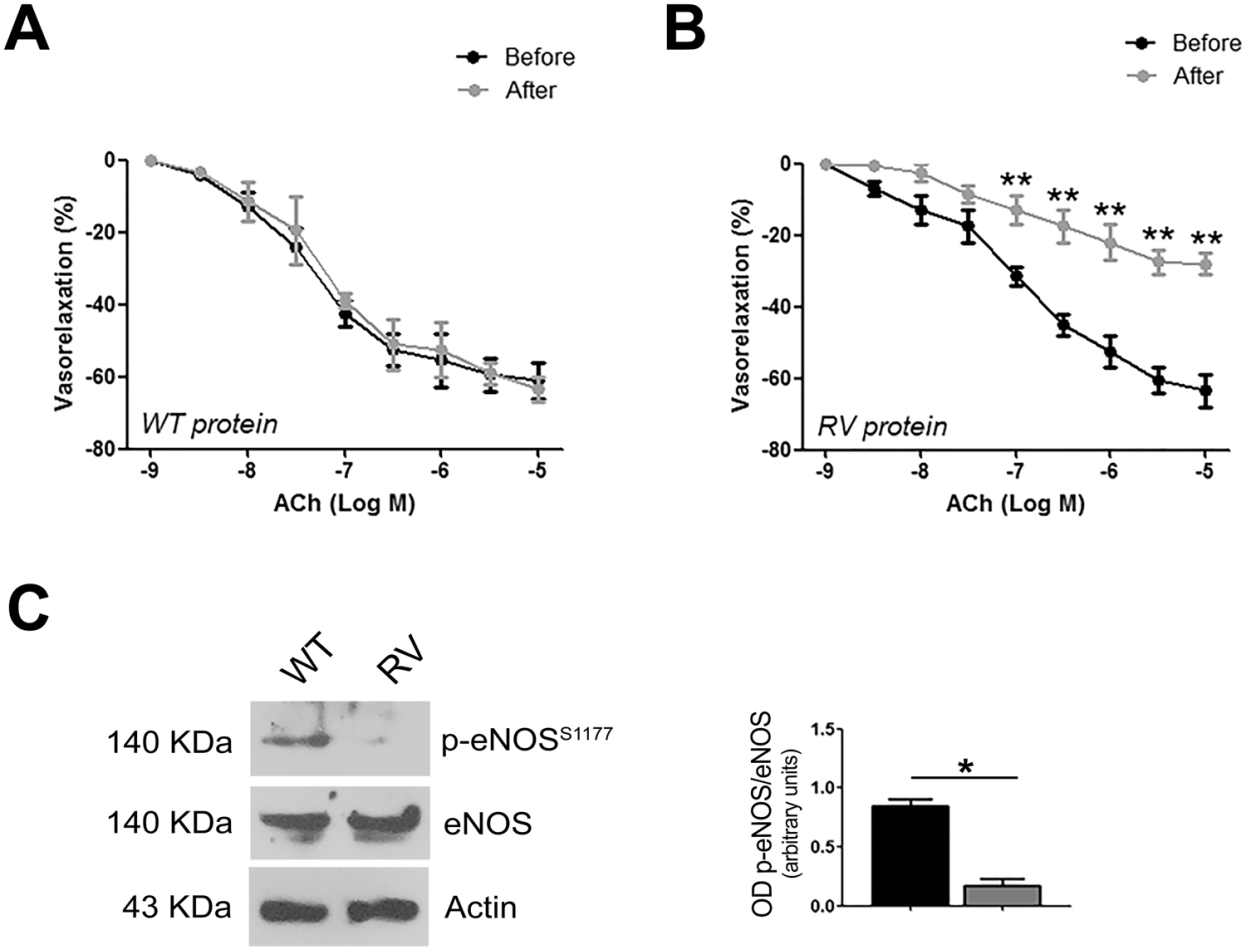
Reaction of *ex vivo* human arteries to exposure to recombinant WT- and RV-BPIFB4 proteins. (**A**,**B**) Dose-response of human superior thyroid arteries (STA) incubated for 1 hour with 18ng/mL of recombinant WT-BPIFB4 (**A**) or RV-BPIFB4 (**B**) protein to increasing doses of acetylcholine (Ach). Values are means ± SEM (n = 4 experiments).**p < 0.01 (two-way ANOVA followed by Bonferroni post hoc test). (**C**) *left*, Representative immunoblot of human STA after vascular reactivity studies; *right*, Columns are the mean ± SEM of 3 independent experiments. *p < 0.05 (Student’s t-test). Images were cropped using Adobe Photoshop, full-length blots are presented in Supplementary Fig. 4.

Exposure to recombinant RV-BPIFB4 protein significantly reduced acetylcholine-evoked endothelial vasorelaxation as compared to WT protein **(**Fig. 3A,B**)**, an effect associated with impaired eNOS phosphorylation **(**Fig. 3C**)**; no effect was observed on vasorelaxation evoked by nitroglycerine (data not shown).

## Discussion

The complex interplay between genes and environmental factors affecting the regulation of blood pressure is not well understood yet. In this regard, cardiovascular medicine is increasingly focused on the search for new risk markers and potential therapeutic targets. Over the last few years, genome-wide association studies have identified more than 50 genetic variants associated with cardiovascular disease and have given valuable insights into the pathological mechanisms underlying specific disorders such as cerebrovascular and coronary artery diseases and myocardial infarction^10, 11^.

Regarding hypertension, only 50% of patients attain good levels of blood pressure despite the wealth of pharmacological treatments available to them. Genetic studies are revealing how the genetic background of these patients influences the response to anti-hypertensive medication^12^. Indeed, the effect of a particular anti-hypertensive drug is influenced not only by the patient’s ethnicity but also by the disease’s genetic etiopathology. Moreover, a complex hypertensive trait – and any correlated inefficacy of treatment – is generated in many cases by an interplay of genetic and environmental factors. There is also a growing body of evidence in favor of an inverse association between genetic factors of exceptional longevity and the risk of high blood pressure: for example, the expression of CAMK4 has been associated with exceptional longevity, whereas its deletion induces hypertension^13, 14^. Continuing along this line, the present study demonstrates that the expression of a rare variant of BPIFB4 – a protein we have previously associated with improved aging when present in its longevity-associated isoform – leads to the maintenance of high blood pressure in the face of treatment for hypertension. Many genes involved in the molecular mechanism of action of drugs have been reported to have polymorphisms that modulate drug efficacy: for example, the Arg389Gly substitution in the beta1-adrenergic receptor is associated with lower therapeutic efficacy of beta-blockers^15–17^.

Our findings on a small cohort of patients candidate RV-BPIFB4 as a possible genetic risk factor for high blood pressure. Thus, we focused our attention on experimental models in order to support the human study, which was performed on a limited number of subjects. First of all, we transfected mouse resistance vessels with plasmids encoding the RV or the WT isoform of BPIFB4 to evaluate the impact on vascular function, finding that RV-BPIFB4 impairs eNOS signaling and induces endothelial dysfunction, hallmarks of aging and cardiovascular disease. The detrimental effect of the RV on eNOS function was associated with impairment of phosphorylation on serine 75 of BPIFB4. This finding is coherent with our previous study demonstrating that phosphorylation of serine 75 on LAV-BPIFB4 has a protective effect on the cardiovascular system, enhancing eNOS function and NO release through recruitment of protein 14-3-3 and HSP90^8^. We then injected RV- or WT-BPIFB4-encoding AAV vectors into mice, evaluating hemodynamic effects and vascular function: forced expression of RV-BPIFB4 in WT mice evoked vascular eNOS dysfunction and increased basal systolic blood pressure to a degree similar to that seen in eNOS-deficient mice.

These studies on experimental models helped us understand the mechanisms underlying the influence of RV-BPIFB4 on cardiovascular homeostasis. To evaluate the significance of these findings for humans, we constructed a human recombinant RV-BPIFB4 protein and tested its effects on human vessels. The exposure of human vessels to this protein led to impairment of NO production and endothelial dysfunction, corroborating the results obtained in the mouse.

NO plays a key role in blood pressure homeostasis. Experimental models of hypertension strongly support this concept since impairment of NO production – either pharmacologically with L-NAME or genetically by inactivation of *eNOS* – increases blood pressure. RV-BPIFB4 hinders NO signaling, generating endothelial dysfunction and hypertension. It is not clear whether endothelial dysfunction – a typical trait of cardiovascular diseases such as arterial hypertension – participates in hypertension or is evoked by high blood pressure. However, our *in vivo* experiments demonstrate that RV-BPIFB4 is involved in the impairment of eNOS function in a setting not influenced by high pressure, so endothelial dysfunction induced by the variant causes increased blood pressure and not vice versa. Therefore, we can assert that RV-BPIFB4 is an important regulator of endothelial nitric oxide function and is a modulator of blood-pressure homeostasis.

## Conclusions

*BPIFB4* was recently identified among the genes contributing to equine evolution^18^. In the light of this, while LAV-*BPIFB4* has been positively selected by evolution in man, RV-*BPIFB4* could be the result of a stochastic recombination event that generated a WT/LAV-*BPIFB4* chimera. Once confirmed in larger and well powered cohorts of subjects, the results of our study could suggest that the early identification of RV-*BPIFB4* carriers could open new therapeutic perspectives for individuals who are predisposed to cardio- and cerebro-vascular diseases related to endothelial nitric oxide synthase dysfunction.

### Study limitations

The present study’s design is not exposed to stratification bias typical of case-control studies. However, since post hoc simulations showed that our analyses reached sufficient statistical power to detect the effect sizes observed in the subset of individuals on therapy (>0.80) but not in the entire cohort (<0.80), studies conducted on larger groups of patients will be needed to better clarify whether RV-BPIFB4 can be regarded as an early marker of endothelial injury associated with hypertension. Large independent cohorts of individuals thoroughly characterized from a phenotypic point of view are therefore needed to confirm the association of this extremely rare haplotype (prevalence = 4%) with relevant variations in blood pressure. Further, the lack of statistically significant differences in terms of BP between LAV and WT haplotypes could be due to the extremely low statistical power reached given the observed effect sizes and sample size (<0.25). Moreover, due to the multifactorial characteristics of high blood pressure, the definition and validation of multivariate risk stratification schema, integrating genetic and clinical information, will allow early identification of patients at high risk of adverse events.

## Methods

A supplemental section for more detailed methods is available online in the Supplementary Information file.

### *Ex vivo* transfection of mouse vessels and evaluation of vascular reactivity

All experiments involving animals conformed with institutional guidelines and were approved by IRCCS INM Neuromed’s review board (1070/2015 PR) and complies with NIH guidelines for care and use of laboratory animals. Second-order branches of the mesenteric arterial tree were removed from C57BL/6 or eNOS-deficient (*Nos3*^*tm1Unc*^) mice and transfected as described previously^19^. Endothelium-dependent relaxation was assessed by measuring the dilatory responses of mesenteric arteries to cumulative concentrations of acetylcholine (from 10^−9^ M to 10^−5^ M) in vessels pre-contracted with U46619 at a dose necessary to obtain a similar level of pre-contraction (80% of initial KCl-evoked contraction) in each ring^20^.

### Infection of mice with AAV and measurements of vascular function and blood pressure

After temporary clamping of the proximal and distal femoral arteries of C57BL/6 or eNOS-deficient mice, either 100 µl of saline alone or saline plus AAV-WT-*BPIFB4* or AAV-RV-*BPIFB4* was infused. Mice were sacrificed four days after surgery and infection. Femoral arteries were excised and placed on a wire system for vascular reactivity studies. Systolic and diastolic blood pressures were measured in another experimental series of C57BL/6 mice by tail-cuff plethysmography, as previously described^21^.

### Patient recruitment

The study was performed on a population of 461 individuals. For each, venous blood (10 mL) was withdrawn for analyses and detailed anamnesis was collected. All participants signed an informed consent for the management of personal anamnestic data and blood samples. The study was approved by the IRCCS MultiMedica ethical committee and conducted in accordance with the ethical principles deriving from the Declaration of Helsinki.

### Genotyping

DNA samples were genotyped with Taqman assays for *rs2070325* and *rs11699009*, to identify the haplotype. Data analysis was performed with QuantStudio software 1.1 (ThermoFisher Scientific).

### Statistical analyses

SNP quality control allowed estimation of the minor allele frequency (MAF) and testing for statistically significant deviations from the Hardy–Weinberg equilibrium. The most likely haplotype phases for each individual were estimated by the–hap command implemented in the PLINK software tool and confirmed by the Beagle v.4.1 software tool^22, 23^. The frequency of each haplotype allele was assessed by custom R scripts (www.r-project.org). As for SNP allele frequency, the haplotype allele frequency was estimated by counting the number of chromosomes carrying a specific haplotype divided by the total number of chromosomes observed.

Since systolic and diastolic blood pressure distributions deviated significantly from normal (Shapiro–Wilk test, p < 0.05), the non-parametric Wilcoxon rank-sum test was applied to evaluate the presence of statistically significant differences in terms of quantitative traits distribution between single SNP genotypes and between haplotype allele configurations (assuming a dominant or recessive genetic model). Multivariate linear regression with stepwise feature selection was employed to adjust the association statistics for the impact of demographical and clinical covariates (sex, age, diabetes, GTT, BMI, triglyceridemia, total and HDL cholesterolemia, anti-hypertensive treatments) representing potential confounding factors. Treatment-specific associations were evaluated by including the SNP/haplotype x treatment interaction term in the linear regression fits.

The Wilcoxon rank-sum test or the Kruskal–Wallis test were applied to compare DBP and SBP distribution by categorical variable values as appropriate. The Spearman correlation test was applied to quantify the strength of the correlation between DBP, SBP, and continuous variables of interest. Statistical genetics analysis was performed with the R statistical software tool (www.r-project.org), except when specified otherwise.

### Cloning and purification of recombinant BPIFB4-His protein

HEK293T cells were transfected with the WT-*BPIFB4* or RV-*BPIFB4* vector cloned in fusion with His-Tag. The recombinant protein was purified using affinity Nuvia IMAC Resin (Bio-Rad) under native conditions.

### Evaluation of recombinant RV-BPIFB4 protein in human vessels

To translate the data obtained in the experimental model, we performed vascular reactivity studies using recombinant protein on superior thyroid artery (STA) removed from patients undergoing carotid revascularization^24^. Vascular reactivity was evaluated as previously described^24^. Vessels were incubated with increasing doses of protein (4.5, 9, and 18 ng/mL), and acetylcholine- or nitroglycerine-mediated vasorelaxation assessed. The experimental protocol was approved by IRCCS INM Neuromed ethical committee and carried out in accordance with the institute’s guidelines; all patients gave their informed consent for STA excision.

## Electronic supplementary material

Supplementary Information

Supplementary Dataset File
