# Author Correction: A rare genetic variant of BPIFB4 predisposes to high blood pressure via impairment of nitric oxide signaling

**DOI:** 10.1038/s41598-019-45691-1

**Published:** 2019-06-28

**Authors:** Carmine Vecchione, Francesco Villa, Albino Carrizzo, Chiara Carmela Spinelli, Antonio Damato, Mariateresa Ambrosio, Anna Ferrario, Michele Madonna, Annachiara Uccellatore, Silvia Lupini, Anna Maciag, Larisa Ryskalin, Luciano Milanesi, Giacomo Frati, Sebastiano Sciarretta, Riccardo Bellazzi, Stefano Genovese, Antonio Ceriello, Alberto Auricchio, Alberto Malovini, Annibale Alessandro Puca

**Affiliations:** 1 IRCCS Neuromed, 86077 Pozzilli, (IS) Italy; 2 Department of Medicine and Surgery, University of Salerno, Fisciano, 84084 (SA) Italy; 3 Cardiovascular Research Unit, IRCCS MultiMedica, 20099 Sesto San, Giovanni (MI) Italy; 4 Institute of Biomedical Technologies, National Research Council, 20090 Segrate, (MI) Italy; 5 University of Milan, Via Festa del Perdono, 20122 Milan, Italy; 6 Department of Translational Research and New Technologies in Medicine and Surgery, University of Pisa, Pisa, 56126 Italy; 7 Department of Medico-Surgical Sciences and Biotechnologies, Sapienza University of Rome, 04100 Latina, Italy; 8 Laboratory of Informatics and Systems Engineering for Clinical Research, Istituti Clinici Scientifici Maugeri, 27100 Pavia, Italy; 9 Department of Electrical, Computer and Biomedical Engineering, University of Pavia, Pavia, Italy; 10 Diabetes Endocrine and Metabolic Diseases Unit, IRCCS MultiMedica, 20099 Sesto San, Giovanni (MI) Italy; 11 Institut d’Investigacions Biomèdiques August Pi i Sunyer (IDIBAPS) and Centro de Investigación Biomedica en Red de Diabetes y Enfermedades Metabólicas Asociadas (CIBERDEM), Barcelona, Spain; 12 Department of Cardiovascular and Metabolic Diseases, IRCCS MultiMedica, 20099 Sesto San, Giovanni (MI) Italy; 13 TIGEM (Telethon Institute of Genetics and Medicine), 80078 Pozzuoli, Italy; 14 Department of Translational Medicine, “Federico II” University, Napoli, Italy

Correction to: *Scientific Reports* 10.1038/s41598-017-10341-x, published online 29 August 2017

In the original version of this Article, Antonio Ceriello was incorrectly affiliated with ‘Diabetes Endocrine and Metabolic Diseases Unit, IRCCS MultiMedica, 20099, Sesto San, Giovanni (MI), Italy’. The correct affiliations are listed below.

Institut d’Investigacions Biomèdiques August Pi i Sunyer (IDIBAPS) and Centro de Investigación Biomedica en Red de Diabetes y Enfermedades Metabólicas Asociadas (CIBERDEM), Barcelona, Spain

Department of Cardiovascular and Metabolic Diseases, IRCCS MultiMedica, 20099 Sesto San Giovanni (MI), Italy

This error has now been corrected in the PDF and HTML versions of the Article, and in the accompanying Supplementary Information file.

