# Supplementary material for: A rare genetic variant of BPIFB4 predisposes to high blood pressure via impairment of nitric oxide signaling
Source: Sci Rep. 2017 Aug 29;7:9706. doi: 10.1038/s41598-017-10341-x (PMC5574984; doi:10.1038/s41598-017-10341-x)
Supplement: Supplementary file 1 — Supplementary Information [file 41598_2017_10341_MOESM1_ESM.doc]

SUPPLEMENTAL MATERIAL

**A rare genetic variant of *BPIFB4* predisposes to high blood pressure via impairment of nitric oxide signaling**

Carmine Vecchione^1,2^*, Francesco Villa^3^, Albino Carrizzo^1^, Chiara Carmela Spinelli^3^, Antonio Damato^1^, Mariateresa Ambrosio^1^, Anna Ferrario^4^, Michele Madonna^1^, Annachiara Uccellatore^5^, Silvia Lupini^5^, Anna Maciag^3^, Larisa Ryskalin^6^, Luciano Milanesi^4^, Giacomo Frati^1,7^, Sebastiano Sciarretta^1,7^, Riccardo Bellazzi^8,9^, Stefano Genovese^10^, Antonio Ceriello^11,12^, Alberto Auricchio^13,14^, Alberto Malovini^8^, Annibale Alessandro Puca^2,3^*

1, IRCCS Neuromed, 86077 Pozzilli (IS), Italy;

2, Department of Medicine and Surgery, University of Salerno;

3, Cardiovascular Research Unit, IRCCS MultiMedica, 20099 Sesto San Giovanni (MI), Italy;

4, Institute of Biomedical Technologies, National Research Council, 20090 Segrate (MI), Italy;

5, University of Milan, Via Festa del Perdono, 20122 Milan, Italy;

6, Department of Translational Research and New Technologies in Medicine and Surgery University of Pisa, Italy;

7, Department of Medico-Surgical Sciences and Biotechnologies, Sapienza University of Rome, 04100 Latina, Italy;

8, Laboratory of Informatics and Systems Engineering for Clinical Research, Istituti Clinici Scientifici Maugeri, 27100 Pavia, Italy;

9, Department of Electrical, Computer and Biomedical Engineering, University of Pavia, Pavia, Italy;

10, Diabetes Endocrine and Metabolic Diseases Unit, IRCCS MultiMedica, 20099 Sesto San Giovanni (MI), Italy;

11, Institut d’Investigacions Biomèdiques August Pi i Sunyer (IDIBAPS) and Centro de Investigación Biomedica en Red de Diabetes y Enfermedades Metabólicas Asociadas (CIBERDEM), Barcelona, Spain;

12, Department of Cardiovascular and Metabolic Diseases, IRCCS MultiMedica, 20099 Sesto San Giovanni (MI), Italy.

13, TIGEM (Telethon Institute of Genetics and Medicine), 80078 Pozzuoli, Italy;

14, Department of Translational Medicine, "Federico II" University, Napoli, Italy;

*, Co-corresponding authors

**Detailed Methods**

**Ex Vivo transfection of mouse vessels and evaluation of vascular reactivity**

All experiments involving animals conformed with institutional guidelines, were approved by IRCCS INM Neuromed’s review board (1070/2015 PR), and complied with NIH guidelines for care and use of laboratory animals. Second-order branches of the mesenteric arterial tree were removed from C57BL/6 or eNOS-deficient (*Nos3^tm1Unc^*) mice and transfected as described previously.^1^ Briefly, vessels were placed in a Mulvany pressure system filled with Krebs solution supplemented with 20 μg of the pRK5 vector encoding either WT-*BPIFB4* or RV-*BPIFB4*, or with an empty plasmid as a negative control. All vessels were perfused at 100 mmHg for 1 h and then at 60 mmHg for 5 h. Endothelium-dependent relaxation was assessed by measuring the dilatory responses of mesenteric arteries to cumulative concentrations of acetylcholine (from 10^−9^ M to 10^−5^ M) in vessels pre-contracted with U46619 at a dose necessary to obtain a similar level of pre-contraction (80% of initial KCl-evoked contraction) in each ring.^2^ Caution was taken to avoid endothelial damage. The corresponding values are reported as a percentage of lumen diameter change after exposure to the substance. Responses were tested before and after transfection. Statistical analyses was conducted using two-way ANOVA followed by Bonferroni post hoc test.

**Infection of mice with AAV and measurements of vascular function and blood pressure**

Femoral arteries of C57BL/6 or eNOS-deficient mice weighing ~25 g were used in this study. The mice were placed individually in an induction chamber and anesthesia induced with 5 % isoflurane in 100 % O_2_ (delivery rate, 5 L/min) until loss of righting reflex. After induction, the mice were placed in dorsal recumbence on a homeothermic blanket (N-HB101-S-402) to maintain body temperature at 37°C. Anesthesia then was maintained with 1% isoflurane in 100 % O_2_ at 1.5 L/min, administered by means of a facemask connected to a coaxial circuit (Fluovac anesthetic mask). The procedures replicated typical clinical practice when laboratory mice are anesthetized for surgical procedures. Vascular surgery was performed with the aid of a microscope at 2–10X magnification. Femoral arteries were exposed and isolated circumferentially from the inguinal ligament to the knee; all side branches were ligated. To obtain an isolated arterial segment, the superficial femoral artery was first controlled proximally with two microvascular clips. After temporary clamping of the proximal and distal femoral arteries, 100 µl of saline alone, of saline plus AAV-WT-*BPIFB4*, or of AAV-RV-*BPIFB4* was infused into the femoral artery and incubated for 15 minutes. Viral titer was 1×10^13^ GC/kg for each experimental condition. After incubation, the distal femoral artery was permanently ligated, and clamps on the proximal femoral artery were removed to restore femoral blood flow. Mice remained anesthetized for 1 h, after which all received 100 % O_2_ until recovery of righting reflex.

Mice were sacrificed four days after surgery and infection. Femoral arteries were excised and placed on a wire system for vascular reactivity studies. Systolic (SBP) and diastolic (DBP) blood pressures were measured in another experimental series of C57BL/6 mice by tail-cuff plethysmography, as previously described.^3^

Data of vessel reactivity are given as mean±standard error of mean (SEM), and were analyzed with Student’s t-test or two-way ANOVA followed by Bonferroni post-hoc analysis, as appropriate, using a dedicated software (GraphPad Prism Software, version 5.0).

**Western blotting**

Western blotting was performed on pooled protein extracts from transfected perfused vessels or from infected mice vessels. Protein extracts were separated on 10 % SDS-PAGE at 100 V for 1 h or on 4–12 % SDS-PAGE at 100 V for 2 h and then transferred to a nitrocellulose or PVDF membranes. The membranes were incubated overnight with the following primary antibodies from Cell Signaling Technology unless otherwise stated: anti-phospho-eNOS Ser1177 (1:1000 rabbit mAb), anti-eNOS (1:800 mouse mAb), anti-phospho-BPIFB4 Ser75 (monoclonal antibody produced in collaboration with Areta International, <http://www.aretaint.com/>),^4^ anti-BPIFB4 (1:200 rabbit pAb, Abcam), anti-beta-actin (1:3000 mouse mAb). After a triple wash, membranes were incubated for 1–2 h with the secondary antibody (1:3000 horseradish peroxidase-linked anti-rabbit IgG or anti-mouse IgG; Amersham). The membranes were then washed four times and specific protein bands detected with ECL Prime chemiluminescent agents (Amersham). Western-blot data were analyzed using ImageJ software (Wayne Rasband, National Institutes of Health, USA) to determine optical density (OD) of the bands. The OD readings of phosphorylated proteins are expressed as a ratio relative to total protein or to beta-actin. All other protein expressions are normalized to beta-actin to account for variations in loading.

Densitometric data were analyzed with Student’s *t*-test or one-way ANOVA, as appropriate, using a dedicated software (GraphPad Prism Software).

**Patient recruitment**

We enrolled 461 individuals (range, 40–75 years old) attending general practitioners of the ASL Città Metropolitana di Milano. Exclusion criteria were: previous diagnosis of type 2 diabetes; serious disease with reduced life expectancy; chronic therapy with drugs with the potential to induce diabetes; evidence or history of drug or alcohol abuse; and severe mental deficiency limiting ability or unwillingness to comply with the study’s requirements.

Venous blood (10 mL) was withdrawn from each individual for the following: genotyping; an oral glucose tolerance test (GTT), assessing plasma glucose at fasting (FPG) and 120 minutes after ingestion of 75 g glucose (2 h PG); advanced glycation end-products (AGE), assessed by skin intrinsic fluorescence (SIF); triglyceridemia; total and high density lipoprotein (HDL) cholesterolemia; and glycated hemoglobin (HbA1c). Systolic and diastolic blood pressures have been measured three times using a sphygmomanometer. Body mass index (BMI), heart rate, disease status, and medical history (including anti-hypertensive treatments) were also determined.

All participants signed an informed consent for the management of personal anamnestic data and blood samples. The study was approved by ethical committee and conducted in accordance with the ethical principles deriving from the Declaration of Helsinki.

**Genotyping**

DNA was extracted from peripheral blood (QIAamp DNA Blood kit, Qiagen) and genotyped with Taqman assays on a QuantStudio 6 Flex real-time PCR System (ThermoFisher Scientific). We used 2 Taqman probes, for *rs2070325* and *rs11699009*, to identify the haplotype of each sample. The reactions were performed using Genotyping Master Mix (ThermoFisher Scientific). Data analysis was performed with QuantStudio software 1.1 (ThermoFisher Scientific).

**Data availability**

All data generated or analyzed during this study are included in this published article and its Supplementary Information files.

**Cloning and purification of recombinant BPIFB4-His protein**

WT-*BPIFB4* and RV-*BPIFB4* coding sequences were PCR-amplified and cloned in fusion with His-Tag in pCDH cloning and expression vector under the EF-1 promoter. HEK293T cells were transfected with appropriate vector using the CaCl_2_ and HBS method. Cells expressing BPIFB4-His protein were harvested after 48 h from transfection and placed in lysis buffer/binding buffer (20 mM sodium phosphate, 300 mM NaCl, 20mM imidazole, pH 8.0). Cell extract was digested with DNasi I (Promega) and sonicated. The recombinant protein was purified using affinity Nuvia IMAC Resin (Bio-Rad) under native conditions, washed, and eluted from the column with elution buffer (20 mM sodium phosphate, 300 mM NaCl, 150–200 mM imidazole, pH 8.0). The quality and purity of BPIFB4-His recombinant protein was checked by Coomassie staining and Western blotting with anti-BPIFB4 (Abcam) and anti-His-tag (Cell Signaling) antibodies.

**Evaluation of RV-BPIFB4 protein in human vessels**

To translate the data obtained in the experimental model, we performed vascular reactivity studies using recombinant protein on superior thyroid artery (STA) removed from patients undergoing carotid revascularization.^5^ The experimental protocol was approved by the local ethical committee and carried out in accordance with the institute’s guidelines; all patients gave their informed consent for STA excision. Vascular reactivity was evaluated as previously described.^5^ Vessels were incubated with increasing doses of protein (4.5, 9, and 18 ng/mL), and acetylcholine- and nitroglycerine-mediated vasorelaxation assessed. Statistical analysis was conducted using two-way ANOVA followed by Bonferroni post hoc test.

**Supplementary References**

1. Vecchione, C. et al. Protection from angiotensin II-mediated vasculotoxic and hypertensive response in mice lacking PI3Kgamma. *J Exp Med* **201**, 1217-1228 (2005).

2. Vecchione, C. et al. Selective Rac-1 inhibition protects from diabetes-induced vascular injury. *Circ Res* **98**, 218-225 (2006).

3. Zacchigna, L. et al. Emilin1 links TGF-beta maturation to blood pressure homeostasis. *Cell* **124**, 929-942 (2006).

4. Villa, F. et al. Genetic Analysis Reveals a Longevity-Associated Protein Modulating Endothelial Function and Angiogenesis. *Circ Res* **117**, 333-345 (2015).

5. Carrizzo, A. et al. Resveratrol improves vascular function in patients with hypertension and dyslipidemia by modulating NO metabolism. *Hypertension* **62**, 359-366 (2013).

**Supplementary Figures**


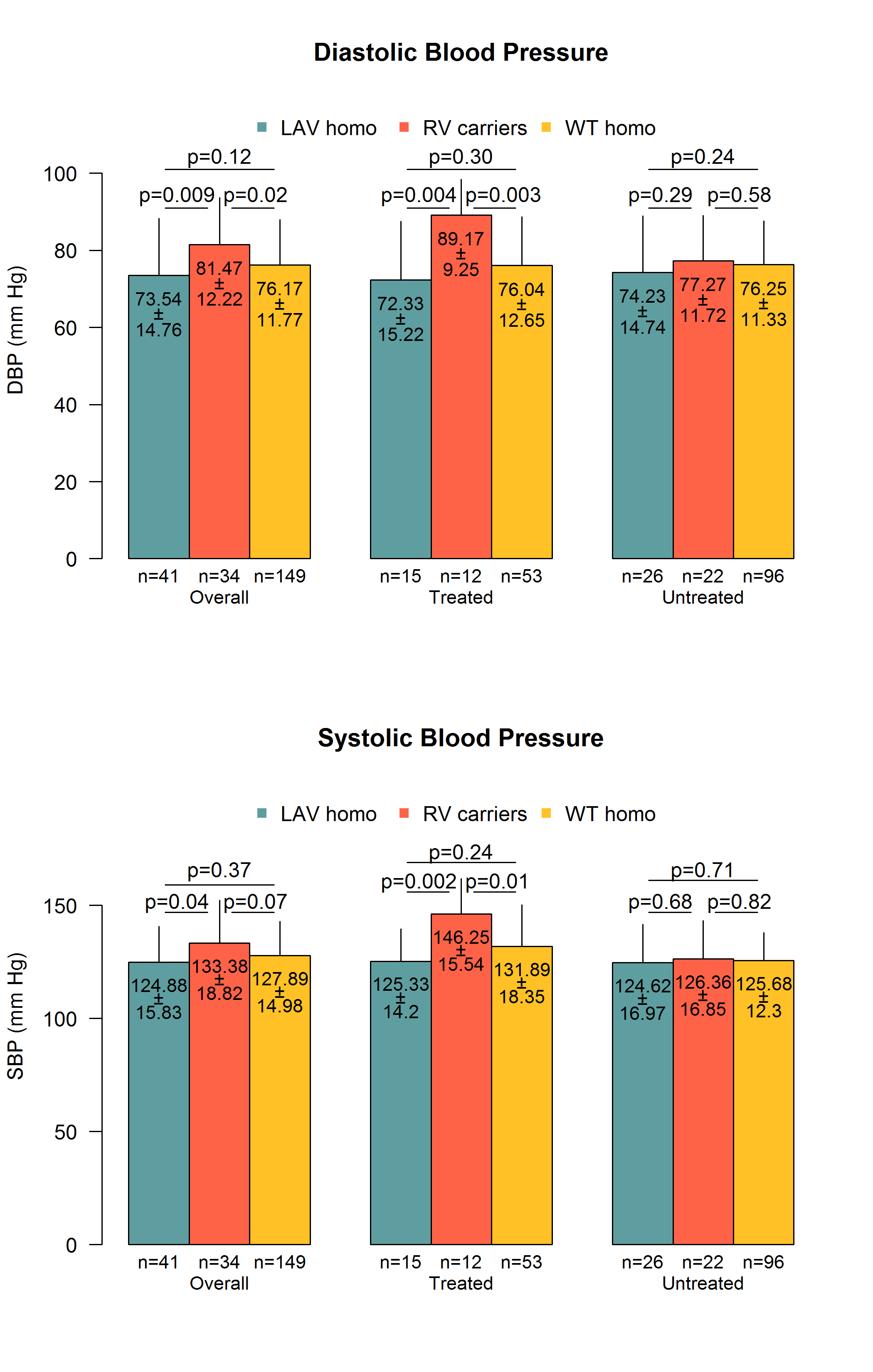


**Supplementary Figure 1. Mean diastolic and systolic blood pressures.**

Blood pressures for LAV homozygotes, RV carriers, and WT homozygotes in the whole cohort (Overall) and in patients stratified on the basis of hypertensive treatment (Treated and Untreated). Bars represent mean value and standard deviation of diastolic or systolic blood pressure by haplotype. p-values were estimated by the Wilcoxon rank-sum test.

**
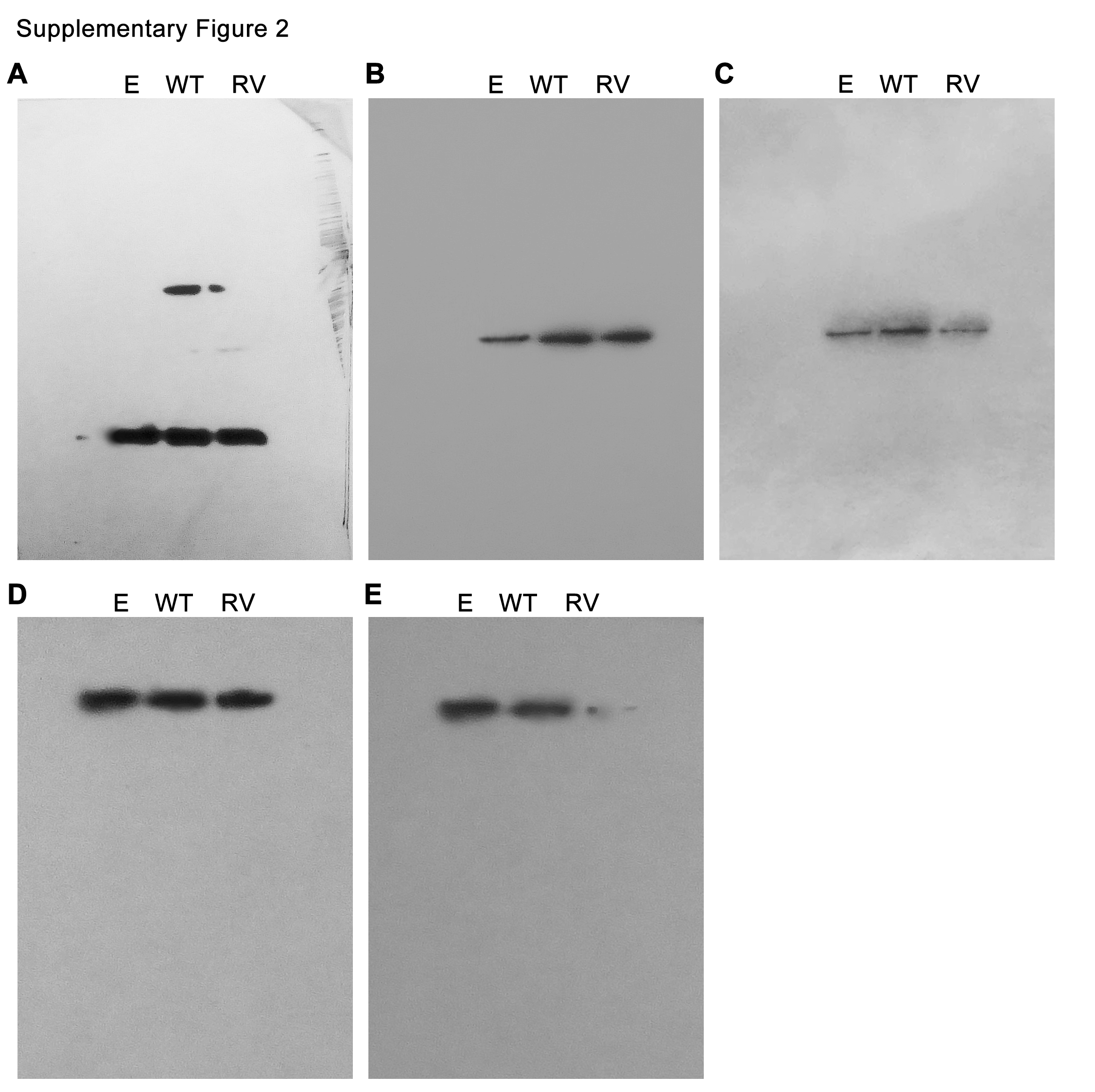
**

**Supplementary Figure 2. Uncropped Figure 1 of main text. A**, Anti β-Actin (8H10D10) Mouse mAb #3700, Cell Signaling. **B**, Anti-C20orf186 (BPIFB4) antibody, ab168171, Abcam. **C**, anti-phospho-Ser75 BPIFB4, 4G3 clone, Areta International. **D**, anti-eNOS Antibody #9572, Cell Signaling. **E**, anti-eNOS (phospho S1177) antibody, Rabbit mAb #9570, Cell Signaling.

**
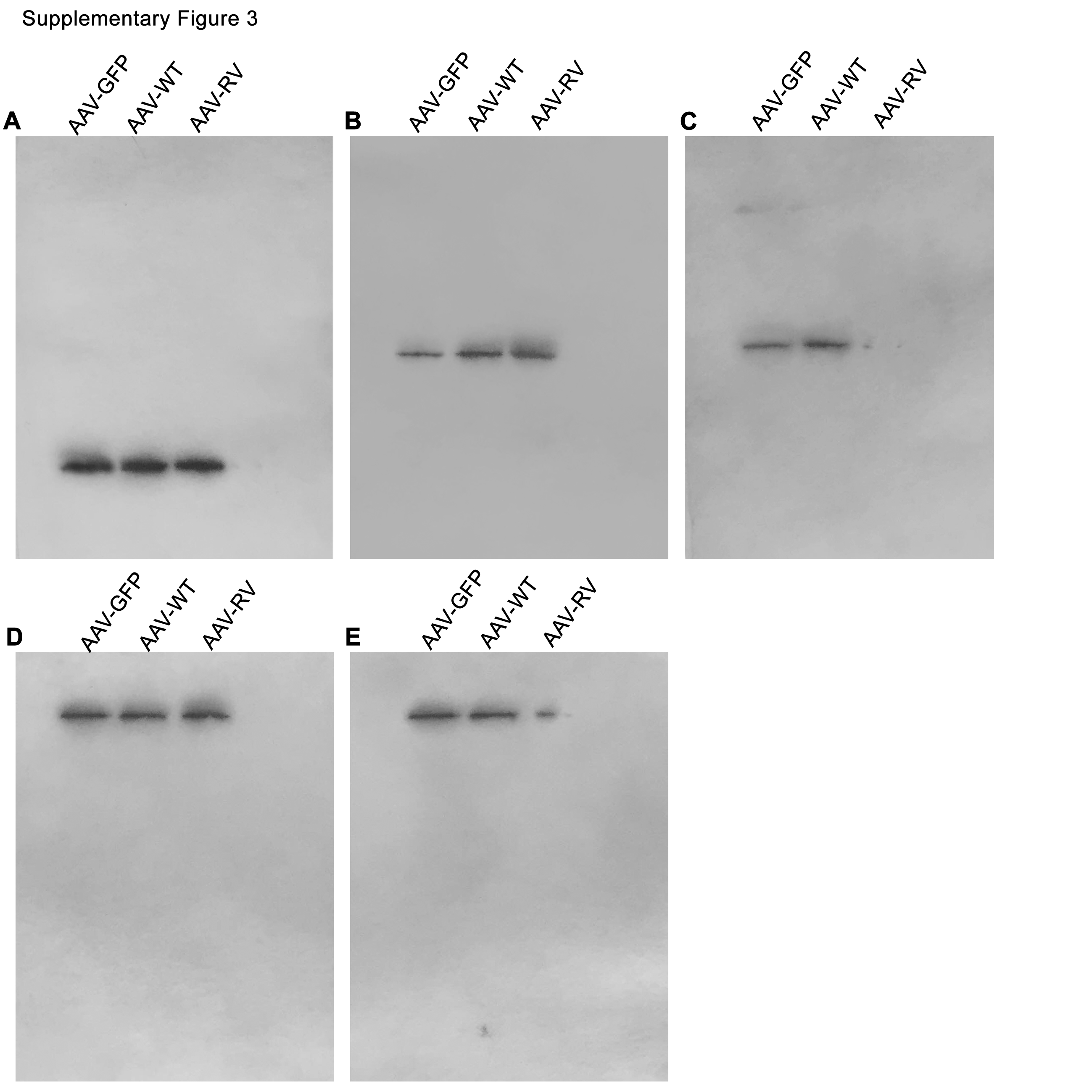
**

**Supplementary Figure 3. Uncropped Figure 2 of main text. A**, Anti β-Actin (8H10D10) Mouse mAb #3700, Cell Signaling. **B**, Anti-C20orf186 (BPIFB4) antibody, ab168171, Abcam. **C**, anti-phospho-Ser75 BPIFB4, 4G3 clone, Areta International. **D**, anti-eNOS Antibody #9572, Cell Signaling. **E**, anti-eNOS (phospho S1177) antibody, Rabbit mAb #9570, Cell Signaling.

**
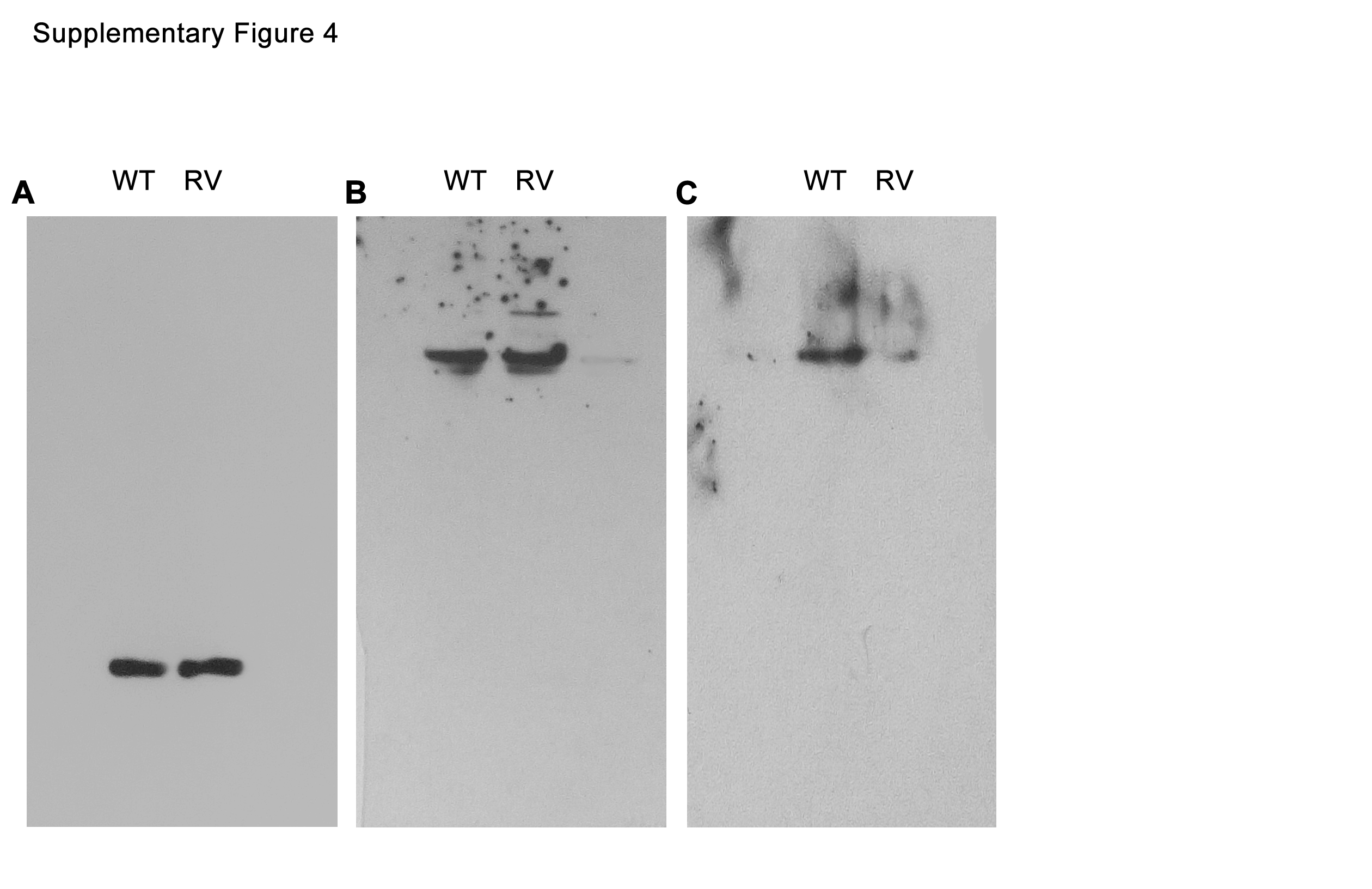
**

**Supplementary Figure 4. Uncropped Figure 3 of main text. A**, Anti β-Actin (8H10D10) Mouse mAb #3700, Cell Signaling. **B**, anti-eNOS Antibody #9572, Cell Signaling. **C**, anti-eNOS (phospho S1177) antibody, Rabbit mAb #9570, Cell Signaling.

**Supplementary Tables**

**Supplementary Table 1.** Univariate associations between variables and diastolic/systolic blood pressure

| **Variable** | **DBP** | |  | **SBP** | |
| --- | --- | --- | --- | --- | --- |
|  | **Value** | **p** |  | **Value** | **p** |
| Sex |  | <0.001* |  |  | <0.001* |
| Males | 80 (70–90); 81.03±11.65 |  |  | 130 (120–140); 132.94±15.02 |  |
| Females | 70 (60–80); 73.44±11.10 |  |  | 130 (110–135); 125.60±14.83 |  |
| Age (years) | + 0.03^#^ | 0.546 |  | + 0.23^#^ | <0.001* |
| Diabetic status |  | 0.614 |  |  | 0.550 |
| Non-diabetic | 80 (70–80); 76.13±11.65 |  |  | 130 (120–140); 128.03±14.79 |  |
| Pre-diabetic | 80 (70–85); 77.16±12.26 |  |  | 130 (120–140); 129.63±15.91 |  |
| Diabetic | 80 (70–90); 77.16±12.22 |  |  | 130 (120–140); 128.68±15.94 |  |
| GTT-FPG | + 0.21^#^ | <0.001* |  | + 0.25^#^ | <0.001* |
| GTT-2HPG | + 0.17^#^ | <0.001* |  | + 0.22^#^ | <0.001* |
| BMI | + 0.28^#^ | <0.001* |  | + 0.25^#^ | <0.001* |
| Triglyceridemia | + 0.16^#^ | <0.001* |  | + 0.10^#^ | 0.029* |
| Total cholesterolemia | – 0.04^#^ | 0.374 |  | – 0.03^#^ | 0.501 |
| HDL cholesterolemia | – 0.21^#^ | <0.001* |  | – 0.16^#^ | <0.001* |
| Anti-hypertensive treatment |  | 0.121 |  |  | <0.001* |
| Yes | 80 (70–90); 77.76±11.67 |  |  | 130 (120–140); 132.34±16.37 |  |
| No | 80 (70–80); 75.88±12.05 |  |  | 130 (120–135); 126.29±14.13 |  |

DBP, diastolic blood pressure; SBP, systolic blood pressure; GTT, oral glucose tolerance test; FPG, fasting plasma glycemia; 2HPG, 2-hour plasma glycemia (after ingestion of 75g glucose); BMI, body mass index; HDL, high density lipoprotein. Distributions are described by median (interquartile range, IQR); mean ± SD or Spearman correlation coefficient (^#^); p = p-value from the Wilcoxon rank-sum test, Kruskal–Wallis test, or Spearman correlation test. * p<0.05.

**Supplementary Table 2.** Impact of the rare variant on blood pressure levels, estimated by multivariate linear regression

|  | **RV carriers vs non-carriers** | | | | |
| --- | --- | --- | --- | --- | --- |
|  | **DBP** | |  | **SBP** | |
|  | **Beta (95% CI)** | **p** |  | **Beta (95% CI)** | **p** |
|  |  |  |  |  |  |
| **Stepwise LR** |  |  |  |  |  |
| Covariates, including therapy (yes/no)* | 4.98 (1.15–8.81) | 0.01 |  | 5.11 (0.22–10.01) | 0.041 |
| Covariates, including therapy (single drugs)** | 5.12 (1.28–8.95) | 0.009 |  | 5.23 (0.36–10.11) | 0.04 |
|  |  |  |  |  |  |
| **All variables LR** |  |  |  |  |  |
| Covariates, including therapy (yes/no)* | 4.99 (1.14–8.84) | 0.011 |  | 5.17 (0.27–10.08) | 0.04 |
| Covariates, including therapy (single drugs)** | 5.08 (1.22–8.94) | 0.01 |  | 5.57 (0.66–10.48) | 0.03 |

DBP, diastolic blood pressure; SBP, systolic blood pressure. Stepwise LR: stepwise linear regression of informative variables including treatments coded as yes/no or as single drugs (a patient may be treated by multiple drugs). All variables LR: multivariate linear regression using all variables including treatments coded as yes/no or as single drugs (a patient may be treated by multiple drugs). *:sex, age, diabetes, glucose tolerance test, body mass index, triglyceridemia, total and high density lipoprotein cholesterolemia, antihypertensive therapy. **:sex, age, diabetes condition, glucose tolerance test, body mass index, triglyceridemia, total and high density lipoprotein cholesterolemia, antihypertensive therapy (ACE inhibitors, beta-blockers, alpha-blockers, angiotensin II antagonists, diuretics, calcium antagonists).

**Supplementary Table 3.** Pharmacological therapy in patients stratified for the presence of the rare variant of BPIFB4

| Drug type | **RV carriers** | **Others** | **P** |
| --- | --- | --- | --- |
| ACE inhibitors | 3 (0.09) | 57 (0.15) | 0.45 |
| Beta-adrenergic blockers | 4 (0.12) | 54 (0.14) | 1 |
| Angiotensin II antagonists | 4 (0.12) | 51 (0.13) | 1 |
| Calcium antagonists | 0 (0) | 30 (0.08) | 0.16 |
| Diuretics | 4 (0.12) | 43 (0.11) | 0.78 |
| Alpha-adrenergic blockers | 0 (0) | 2 (0.01) | 1 |

RV, rare variant. Data indicate number (fraction) of patients.

Please note that patients may have been taking more than one drug type.
